# A Systematic Process to Accurately Link Large-Scale Research Consents to State Public Health Newborn Screening Samples

**DOI:** 10.3390/ijns12020023

**Published:** 2026-04-14

**Authors:** Emily Cheves, Hannah E. Frawley, Angela You Gwaltney, Ana N. Forsythe, Samantha Scott, John Colin Mathews, Jake Dibble, Tanya Reeve, Vesselina Bakalov, Manisha Dass, Heidi L. Cope, Curt Scharfe, Holly Peay

**Affiliations:** RTI International, Genomics and Translational Research Center, Research Triangle Park, 3040 East Cornwallis Road, Durham, NC 27713, USA; hfrawley@rti.org (H.E.F.); agwaltney@rti.org (A.Y.G.); aforsythe@rti.org (A.N.F.); sjscott@rti.org (S.S.); cmathews@rti.org (J.C.M.); treeve@rti.org (T.R.); vbakalov@rti.org (V.B.); mdass@rti.org (M.D.); hcope@rti.org (H.L.C.); cscharfe@rti.org (C.S.); holly.peay@faegredrinker.com (H.P.)

**Keywords:** newborn bloodspot screening, genomic newborn screening, newborn screening, decision tree, team decision making

## Abstract

Research programs can interface with public health programs to generate innovation, yet it is critical to ensure processes that support research activities without infringing on protected data. Genomic newborn screening (gNBS) research programs require reliable methods to link parental consents to the correct newborn screening (NBS) specimen. Early Check is a gNBS research program in North Carolina that uses the residual dried bloodspot (DBS) samples stored at the North Carolina State Laboratory of Public Health (NCSLPH) to screen babies for serious health conditions. Early Check created a systematic approach to match research consents with NBS DBS samples utilizing a fuzzy matching algorithm and manual review of prospective matches utilizing a decision tree. Between 28 September 2023, and 10 June 2025, Early Check received parental consents for 4279 newborns. Of those, 614 (14%) had discrepancies that required further review. More than half of these (349, 57%) required outreach to the consenting parent to resolve differences in information such as name, infant sex, or contact details. The use of probabilistic matching, a decision tree, and structured staff review provides a feasible approach for accurately identifying samples from consented NBS participants.

## 1. Introduction

There are a growing number of newborn screening research programs across the globe, many of which are using the residual newborn screening (NBS) dried blood spot (DBS) samples for additional screening under a consented model [1,2]. Residual DBS are left over after state newborn screening is done to identify a panel of serious and treatable conditions. Research studies can identify ways to expand or improve existing public health practice, including NBS. To make these studies practicable and ethical, feasible methods are needed to quickly and accurately link research consents to NBS DBS data and samples. To our knowledge, no research programs have published their methods to achieve such linkage.

Early Check is a voluntary genomic newborn screening (gNBS) research program in North Carolina that, in collaboration with the North Carolina State Laboratory of Public Health (NCSLPH), uses residual NBS DBS samples to screen for approximately 200 genetic conditions and the risk for developing type 1 diabetes [3,4]. Early Check relies on two prospective datasets from separate systems that do not share a common unique identifier: the NCSLPH NBS demographic records and Early Check electronic consent records.

This matching process between Early Check’s consent records and the correct residual NBS DBS at NCSLPH is complicated by potential discrepancies between the two datasets, which may result from changes in the mother/birthing person’s name, address, or other personal information, as well as data entry errors in one or both datasets. Further, the matching process required the research team to briefly have access to identifiers from mothers/birthing persons who had not consented to enroll their newborns in Early Check. The burden of accuracy was on the study team to ensure that only enrolled newborns are screened, and that no samples from unenrolled newborns are included. Early Check developed an efficient method to link records across these datasets and support the integrity of participant screening. The purpose of this report is to share knowledge on methodologies in matching state public health laboratory NBS DBS sample data sets to electronic consents for research.

## 2. Materials and Methods

State newborn screening is conducted on newborns. DBS are thus from newborns who cannot consent to their own research participation in Early Check. Consent must be provided by a legal guardian. The data held by the NCSLPH for newborns they screen includes basic demographics for the birth mother/birthing parent such as name and address (Table 1 shows information that the NCSLPH uploads into their Laboratory Information Management System (LIMS)).

**Figure 1:**
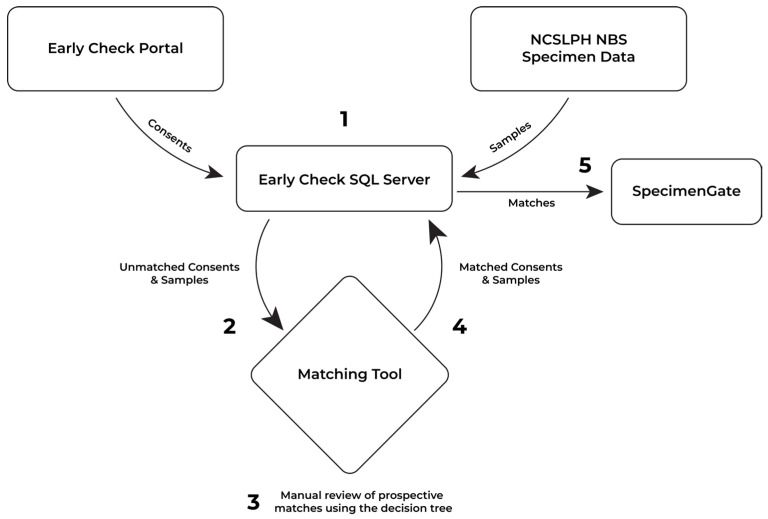
Workflow for matching Early Check consent records and NCSLPH NBS specimen data. (1) Consent records from the Early Check web-based portal and the NCSLPH NBS specimen data are imported into the Early Check SQL Server. (2) Unmatched consents and scored records are exported to the web-based Matching Tool, which includes an embedded fuzzy-matching algorithm that assigns a link probability for each potential consent–specimen pair. (3) Research team members manually review each candidate pair using a standardized decision tree to confirm prospective matches. (4) Confirmed matches are returned to the Early Check SQL Server. (5) Verified consent–specimen pairs are transferred to the SpecimenGate^®^ system for specimen retrieval and genomic newborn screening.

For Early Check, interested mothers/birthing parents used a self-directed web-based portal (https://portal.earlycheck.org), available in both English and Spanish, to learn more about Early Check and enroll their newborns. For scenarios such as adoption, foster care, or gestational carriers, the legal guardian could complete the enrollment, but this required additional outreach to the study team (Early Check requested legal proof of guardianship). Eligible newborns were born in North Carolina, received standard NBS, and were ≤31 days old. Parents could withdraw their baby from Early Check at any time. Early Check’s electronic consent was designed to collect demographic information (Table 1) in a way that aligned with information collected by the state. This provides an opportunity to match data from the mother/birthing parent in the NCSLPH NBS demographic records with data provided at time of research consent by the birth mother. Early Check developed a methodology to link research consents to the NBS DBS sample at the NCSLPH (Figure 1). The study protocol and electronic consent were reviewed and approved by the University of North Carolina Institutional Review Board (IRB; #18-0009; 31 January 2023).

### 2.1. Matching Algorithm

Early Check developed a proof-of-concept application for matching records through a probabilistic or fuzzy matching algorithm [5]. The matching algorithm was applied to five identifying fields shared between the two datasets: first name, last name, address, email address (when available), and normalized telephone number (e.g., removing punctuation and country codes). Potential matches between consent records and specimen records were identified using a fuzzy string-matching procedure based on the Fuse.js library (v6.4.6). Each consent record was compared against candidate NCSLPH specimen records retrieved within a predefined temporal window of ±105 days surrounding the infant’s date of birth (or due date when birth date was unavailable). The objective was to quantify the degree of similarity between identifying fields in the two data sources and to compute a composite match score reflecting the overall likelihood of correspondence.

Probabilistically matched records were displayed through a custom software application built in a HIPAA-compliant and FIPS 140-2 certified secure environment to ensure that all data remained protected during review of each prospective match. Since this matching process required the research team to briefly have access to identifiers from all birthing mothers in the state, including those who had not consented to enroll their newborns in Early Check, data protection was critical. The Early Check consent records provided by the mother/birthing parent through an electronic consent portal were migrated to an SQL Server database (Redmond, WA, USA), a relational database management system (RDBMS) that provides an integrated environment for storing and managing data. The fuzzy matching process evaluated how closely consent records matched NCSLPH NBS specimen card demographic data, assigned a match score, and stored the results in the SQL Server database (Table 2).

Fuse.js computed a similarity/distance score $f_{i}\in \left[0,\;1\right]$ for each $f_{i}\leq 0.1$ consent–specimen field comparison, where 0 indicates an exact match and 1 indicates no similarity. To emphasize stronger identifiers, fields were weighted as follows: full name (0.2), street address (0.4), email address (0.7), and home and cell phone numbers (0.8 each). Weighted scores were combined into a single weighted average match score per query, and only candidates with (≥90% similarity) $f_{i}\leq 0.1$ were retained (Table 3). Each retained comparison score was transformed and adjusted so that each fuzzy match contributed a positive score with a max score of 10 if the field is a perfect match. The transformed score from the five fields (i.e., first name, last name, address, email, and phone) were summed to form a base score. Additional deterministic bonuses were applied by adding +1 for an exact phone match, +1 for an exact email match, and +0.1 for identical infant dates of birth, producing the final score. For each consent record, candidate specimens were ranked with higher scores indicating greater similarity and higher confidence of a match. 

### 2.2. Matching Tool

**Figure 2:**
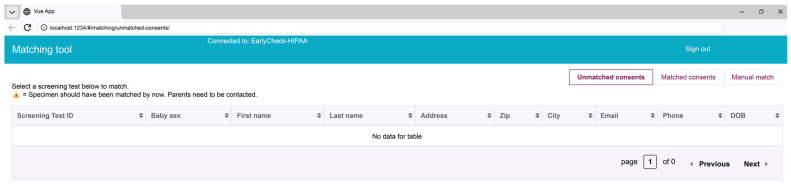
Matching Tool.

Records with a high degree of probabilistic match were then presented to a member of the research matching team through a web-based application referred to as the “matching tool” (Figure 2). The matching tool displayed consents of all babies enrolled in Early Check who had not yet been manually “matched” to a corresponding NBS card sample by a research team member. The interface pulled key information including the mother/birthing parent’s name, address, phone number, email, sex of the newborn, and baby’s date of birth from the electronic consent and presented these variables for review. A member of the matching team clicked on each consent in the matching tool, and the matching algorithm searched the table of NCSLPH NBS demographic records from their LIMS system from the past 6 months to present prospective scored matches to the research team member. Consents not matched within two weeks of date of birth were flagged by the matching tool, and the research team would contact the consenting parent if a specimen could not be found. Matched consents that were reviewed and confirmed by the research team were imported once a day into SpecimenGate^®^ (Revvity, Waltham, MA, USA), a specialized software platform designed specifically for NBS laboratories and used for NBS operations (including instrumentation, lab workflow, patient reporting, and follow-up).

### 2.3. Decision Tree

**Figure 3:**
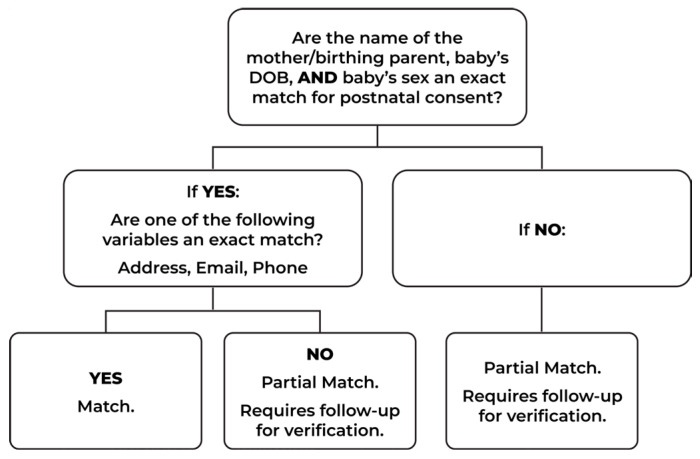
The Decision Tree. The Decision Tree guides manual confirmation of prospective matches. Reviewers assess agreement across key identifiers (mother/birthing parent name, baby’s date of birth, baby’s sex, and fourth variables) to classify each case as a match or a partial match requiring follow-up for verification.

Decision trees are a systematic way to support decision making in situations of uncertainty by implementing a hierarchical tree structure that follows an algorithm [6]. Decision trees are used in training software as well as clinical decision making for scenarios of uncertainty or nuance. Early Check used a fast-and-frugal, purposefully designed decision tree (Figure 3) to visually represent the steps and decisions needed for matching team members to ensure compliance with regulations and policies, most specifically HIPAA requirements and the IRB-approved study protocol, and in accordance with our agreements with the NCSLPH. The decision tree facilitated cross-team consistency, ensuring the integrity of the research study, and reduced the decision-making burden in the manual part of the matching process. The team developed a detailed Standard Operating Procedures (SOP) manual with case scenarios for application of the decision tree. All matching team members were HIPAA certified and completed a required annual HIPAA training due to the sensitive nature of the data.

The decision tree, developed in collaboration with the NCSLPH, prioritized specific identifying variables between the NBS card and Early Check electronic consents to create standards for ensuring the correct sample was matched to the research study participants. Early Check research team members manually reviewed each prospective match using a standardized decision tree to ensure accuracy before any sample was screened (Figure 3). To confirm a match, at least four out of the six identifiers in the matching tool interface had to align between the NCSLPH specimen record and the consent record (Figure 2). Of these, there are three variables the decision tree required to be exact matches: mother/birthing parent’s name, baby’s date of birth, and sex of the baby. To proceed with an acceptable manual match, a fourth variable needed to be confirmed which could be the phone number, email, or mailing address. Often, NBS cards, completed by the hospital/birthing center, are missing one or more of these variables (e.g., email has a low completion rate on the NBS DBS sample card, so that has rarely been used as an identity confirmation for Early Check). The matching team confirmed exact matches once the prospective matches were in the matching tool utilizing the manual decision tree processes.

### 2.4. Discrepancies

Different types of discrepancies could prevent matching (Table 4). Discrepancy resolution could require identity confirmation, laboratory coordination, investigation into databases for a missing specimen, or contacting the research team’s informaticians if there was a technical error.

### 2.5. Identity Confirmation

Identity confirmation discrepancies occurred when identifying information on the consent differed from the NBS card and did not meet the criteria defined by the decision tree. The matching tool only displayed the required identity confirmation variables necessary for matching (Table 1). Some discrepancies were resolved by an Early Check team member by looking directly at the NBS card information in the SQL Server database, such as baby’s sex being written under the baby’s name due to human error. An identity confirmation discrepancy often required outreach to the consenting parent. If no response was received from the consenting parent, the discrepancy could not be resolved, and the participant was withdrawn from the study. There were specific SOP in place for contacting the parent, which included email templates, phone scripts, timelines, and specific procedures for each type of discrepancy scenario.

The research team had definitions of acceptable exact matches that go beyond the scoring system after the matching tool scores for each prospective match. An exact match for the matching team included an obvious or acceptable error on the discrepant identifier. For example, an exact match for a first name could be a common nickname, misspelling (e.g., “Jenniferr” instead of “Jennifer”), or mistype error (e.g., two numbers switched on the street address), that the algorithm determined not as an “exact match”, but the research team defined as acceptable based on their SOP, when considered with the totality of the identity data.

### 2.6. Inputting and Technical Errors

Inputting errors included creating multiple accounts or duplicate consents for an individual baby. In some cases of multiple births (e.g., twins, triplets), the parent created a unique consent for each baby, rather than (as directed on the site) indicating the existence of multiple births during a single consent process. In addition, there were cases where a parent only had one child but mistakenly indicated they had multiple births, or the parent had multiple births but did not indicate that in the consent. Multiple accounts could occur due to parent error but also due to system errors in the Early Check consent system, such as when signing up a second child with Early Check or other factors (e.g., using a different email address). System related errors were mitigated as they were discovered to help reduce the number of technical issues in the future.

### 2.7. Missing or Difficult to Locate Specimens

Specimens in the state laboratory could appear to be missing due to discrepant information on the NBS card, such as the wrong date of birth listed for the baby, or because the quality standards required for accurate and reliable newborn screening were not met (i.e., “Unsatisfactory”) and another NBS DBS sample was needed.

### 2.8. Repeat (Multiple) Specimens per Baby

Repeat specimens occurred when more than one NBS DBS sample was taken for each baby. When the matching tool indicated the existence of multiple specimens for an individual baby the research team had the opportunity to use highest quality specimen for sample collection to match to Early Check electronic consents.

## 3. Results

Early Check received signed consents for 4279 newborns between 28 September 2023, and 10 June 2025. Only 14% (614) had discrepancies that required further review and prevented immediate matching (Table 5). Of those that required outreach for verification, the most common discrepancy was in identity information (349, 57%), and 42 (7%) had greater than one discrepant identity variable. The most frequently discrepant information was the mother/birthing parent’s name, followed by the newborn’s sex and the mother’s address. Discrepancy in phone numbers was rare, and in no case was there discrepancy in email addresses (though email addresses were rarely available in the state database).

Approximately 193 (31%) of the discrepancies were due to repeat specimens for a single baby. These discrepancies required Early Check staff to conduct outreach to the NCSLPH staff to determine the best sample to use for Early Check screening. A subset of cases with multiple samples also had discrepancies in identity variables.

Other issues that prevented matching were rare: technical errors and missing specimens (see Table 5). The matching team withdrew 28 (5%) participants due to inability to confirm identity after staff outreach to the consenting parent.

The median time to match a discrepant consent was 2 days, with 313 (51%) consents matched in 2 days or less, while 108 (18%) of consents required more than 1 week to match the correct specimen, primarily due to delays in parental response (Table 6).

## 4. Discussion

There is growing attention to the ethical, privacy, and policy implications of using residual dried blood spot samples for research as genomic newborn screening (gNBS) programs expand [7]. A foundational requirement for responsible implementation is the ability to accurately link parental consent records to the correct newborn screening specimen with a high degree of accuracy. Misclassification can undermine data integrity and erode public trust in NBS, which remains one of the most successful public health programs [8]. Therefore, it is vital that only consented newborns are screened in research studies. Parents need clear information about what they are consenting to and reassurance that their baby’s data will be protected and handled appropriately in research.

This publication provides one example of the methodology used to match research electronic consents to babies’ DBS. Descriptions of such methods and their outcomes are needed to inform the growing numbers of large-scale newborn screening research studies using DBS specimens. Examples include the newly announced multi-state BEACONS initiative to assess the feasibility of implementing gNBS into public health NBS programs [9] and the Florida Sunshine Genetics Act passed in July 2025, which is a statewide voluntary gNBS program, to identify serious but treatable conditions in the newborn period [10]. Both of these programs will need methods for accurately matching research consents to NBS DBS samples. Through Early Check, a single-state research study, we have shown that discrepancies between consent data and NBS data are not uncommon. This will be increasingly complex for studies that enroll across multiple states. Technology is rapidly increasing with the incorporation of AI and novel programming methodologies. AI has a potential role in efficient and accurate matching of datasets in the future for research in NBS.

Coordinating with state public health laboratories to match DBS screening records to the correct baby is essential to ensure accurate results, protect families’ trust in research, and avoid screening individuals who did not consent. As more large-scale gNBS studies emerge, efficient data-sharing systems and transparent methods that safeguard privacy, consent, and quality control while minimizing burden on public health laboratories and research teams are increasingly important.

Future research should incorporate multiple states for examining the feasibility of implementing quality systems for matching research participants to state NBS programs. A limitation to this research is that Early Check only screens for babies born in North Carolina. Other states and public health laboratories may have different procedures (e.g., NBS card information, laboratory systems). We did not track the type of discrepancy resolution used for participants (e.g., phone call or email contact). The best way to communicate with new families should take into consideration how participants respond in this early newborn period and the ways in which parents typically communicate today (e.g., text). Additionally, Early Check was limited to only contacting the consenting mother/birthing parent or legal guardian listed on the NBS card for confirming discrepant information limiting the ability of other caregivers to help resolve these issues. Many newborns have more than one caregiver or are in the care of a legal guardian (e.g., adoption, surrogacy). In North Carolina, only the mother/birthing parent is listed on the NBS card, limiting the ability for a coparent or non-birthing guardian to enroll the baby or respond to identity confirmation requests from the research team. Including a second legal guardian on the NBS card could be effective in increasing responsivity of caregivers to public health officials and research team members. Contacting the caregivers efficiently is imperative in early identification and intervention for NBS conditions that are more serious.

## 5. Conclusions

The use of probabilistic matching, a decision tree, and structured staff review provides a feasible approach for accurately identifying samples from consented NBS participants. The system narrows potential matches between a large state dataset and a smaller project dataset. This process enables the team to reduce the chance of a mismatch, use a standardized approach with quality control and lower staff burden, and monitor interface performance. Systematic processes such as these are essential for consented NBS research programs that rely on residual DBS following routine NBS.

## Figures and Tables

**Table 1:** Information collected on NCSLPH NBS card and Early Check consents with variables used for matching indicated.

Variable	NCSLPH NBS Card	Early Check Consent	Matching Requirement
Mother/Birthing Parent’s Name	X	X	Exact Match ^1^
Email Address	X	X	Exact Match ^2^
Phone Number	X	X	Exact Match ^2^
Mailing Address (City, State, Zip code, Street Address)	X	X	Exact Match ^2^
Newborn’s Sex	X	X	Exact Match ^1^
Newborn’s Date of Birth	X	X	Exact Match ^1^
Indication if baby is a Multiple (e.g., twins)	X	X	Match Multiples at the same time
Newborn’s Race/Ethnicity	X	X	Not used for matching
Newborn’s Health Care Provider	X	X	Not used for matching
Type of Collection (Initial/Repeat Specimen)			Lab team determines the highest quality specimen to match
Mother/Birthing Parent’s Race/Ethnicity		X	Not used for matching
Mother/Birthing Parent’s Date of Birth		X	Not used for matching
Alternate contact information		X	Not used for matching
Preferred language		X	Not used for matching
Education		X	Not used for matching

^1^ Indicates a required exact match variable to proceed with matching for Early Check. ^2^ Indicates one of three variables that need to be exact to proceed with matching as a fourth variable. Other NBS sample card variables: Hospital Specimen Submitter, Ordering Physician’s name, Type of feeding, Meconium Ileus, Hearing screening, Gestational age, Collection Date/Time, Birthweight.

**Table 2:** Example calculation of fuzzy match score.

Consent Field	Specimen Field	Fuse.js Distance [Equation]	Interpretation
“Jane Doe”	“Jane Doe”	0.00	Perfect match
“Jane Doe”	“Janet Doe”	0.07	Very similar
“Jane Doe”	“Joan Do”	0.25	Moderate similarity
“Jane Doe”	“Maria Lopez”	1.00	Completely different

**Table 3:** Example of weighted similarity calculation for Fuse.js composite match score.

Key	Weight	Per-Key Distance	Weighted Contribution
Mother Full Name	0.2	0.30	0.06
Mother Email Address	0.7	0.02	0.014
Mother Phone	0.8	0.00	0.000
Totals	1.7		0.074/1.7 ≈ 0.044

**Table 4:** Types of discrepancies.

Identity Confirmation	
Name ^1^	Name discrepancies can include an entirely different name or just a different first/last name. A different name can be on the consent in scenarios of adoption, legal guardianship, gestational carriers, or if the father/non-birthing parent filled out the consent.
Date of Birth (DOB) ^1^	Participant DOB on the NBS card and consent portal are different.
Sex ^1^	Differences between the recorded sexes on the consent and sample, or a designation of unknown or “U” on the NBS card.
Mailing Address ^2^	Address discrepancies contain different fields for the street address, city, state, and zip code. Discrepancies are defined for what is acceptable for each of the fields in a mailing address. Street address, city, state, and zip code are required to be the same unless otherwise defined if there appears to be a clear mistake.
Phone Number ^2^	Participant phone numbers are different between the NBS card and consent unless it is a clear mistake (e.g., the same numbers are switched and next to each other on the keyboard).
Email Address ^2^	Participant email is different on the consent than the NBS card.
Technical Errors	Technical errors are scenarios that multiple consents are created due to human or Early Check consent database technical error.
Missing Specimens	Consents that do not have a sample shown in the tool and are past two weeks from the baby’s DOB.

^1^ This variable is a required exact match. ^2^ Only one of these variables is required as an exact match.

**Table 5:** Discrepancies between data on the newborn screening card and study consent data observed in Early Check.

	N = 614	%
**Identity Confirmation** (Require outreach to parent)	349 ^1^	57%
Name	143	23%
Date of Birth	34	6%
Sex	111	18%
Address	86	14%
Phone	22	3.6%
Email	0	0%
**Repeat Specimens** (Require outreach to lab; resolved in 1−2 weeks by lab team)	193 ^2^	31%
**Missing Specimen**	22	3%
**Technical Error**	57	9%
**Withdrawn (Unresolved)** (2 weeks from initial point of contact)	28	5%

^1^ A subset (*n* = 42; 7%) of identity confirmation discrepancies had >1 variable discrepant ^2^ A subset (*n* = 12; 6%) of repeat specimens also had identity confirmation discrepancies.

**Table 6:** Time for Identity Confirmation Resolution.

	Mean Days (SD)	Median Days	Max Days
**Identity Confirmation Discrepancies** (Require reach out to parent)	4.8 (6.7)	2	47
Name	5.2 (7.5)	3	47
Date of Birth	8.9 (9.4) ^1^	5	47
Sex	3.6 (4.3)	2	21
Fourth Variable (Phone number, Email, and Address)	4.3 (5.6)	2	22

^1^ DOB discrepancies took longer to resolve because they would often start out appearing as a missing specimen.

## Data Availability

All data is available in this article.

## Abbreviations

The following abbreviations are used in this manuscript:

gNBS	Genomic Newborn Screening
NBS	Newborn Screening
DBS	Dried blood spots
NCSLPH	North Carolina State Laboratory of Public Health
LIMS	Laboratory Information Management System
SOP	Standard Operating Procedures
RDBMS	Relational Database Management System
NC	North Carolina
